# Digital Health Literacy and Its Role in Awareness of and Access to Sexual Health Products and Services Among Displaced Youth in Uganda’s Informal Urban Settlements: Community-Based Cross-Sectional Study

**DOI:** 10.2196/78343

**Published:** 2025-12-31

**Authors:** Moses Okumu, Carmen Hellen Logie, Isaac Koomson, Thabani Nyoni, Joshua Muzei, Bonita B Sharma, Flora Cohen, William Byansi, Michelle G Thompson, Joseph Cedrick Wabwire, Catherine Naluwende Nafula, Robert Hakiza, Peter Kyambadde, Liliane Cambraia Windsor

**Affiliations:** 1 School of Social Work University of Illinois Urbana-Champaign Urbana, IL United States; 2 School of Social Sciences Uganda Christian University Mukono Uganda; 3 Factor-Inwentash Faculty of Social Work University of Toronto Toronto, ON Canada; 4 United Nations University Institute for Water, Environment, and Health (UNU-INWEH) Richmond Hill, ON Canada; 5 Centre for the Business and Economics of Health The University of Queensland St Lucia Australia; 6 School of Social Work, Faculty of Health Dalhousie University Halifax, NS Canada; 7 Informatics Programs, School of Information Science University of Illinois Urbana-Champaign Urbana, IL United States; 8 Department of Social Work College of Public Policy The University of Texas at San Antonio San Antonio, TX United States; 9 School of Social Work Boston College Chestnut Hill, MA United States; 10 College of Health Lehigh University Bethlehem, PA United States; 11 Department of Health and Kinesiology College of Applied Health Sciences University of Illinois Urbana-Champaign Urbana, IL United States; 12 AVSI Foundation Arua Uganda; 13 YARID: Young African Refugees for Integral Development Kampala Uganda; 14 AIDS Control Program Ministry of Health Kampala Uganda; 15 Most At Risk Populations Initiative Kampala Uganda; 16 Jack, Joseph and Morton Mandel School of Applied Social Sciences Case Western Reserve University Cleveland, OH United States

**Keywords:** digital health literacy, digital sexual health interventions, sexual health equity, refugees, Uganda, gender digital divide.

## Abstract

**Background:**

Digital health interventions can enhance sexual health equity among marginalized and underserved populations, including displaced youth. However, there is limited understanding of displaced youth’s digital health literacy (DHL) and its association with knowledge of and access to sexual health products and services.

**Objective:**

This study aims to identify patterns of DHL among displaced youth and assess how these patterns are associated with awareness of and access to sexual health products and services, while considering gender differences.

**Methods:**

We conducted a cross-sectional tablet-assisted survey in Kampala, Uganda. We used peer-driven sampling to recruit displaced youth aged 16-24 years living in 5 informal urban settlements. We identified DHL patterns using latent profile analysis. Gender-disaggregated multivariate probit models were constructed to estimate the relationship between DHL and awareness of and access to sexual health products and services (eg, sexual and reproductive health [SRH] information, external condoms, condom use training, sexually transmitted infection testing, and HIV testing).

**Results:**

Among the participants (N=445), our latent profile analysis identified 4-DHL classes named: low (class 1, 51/444, 11.5%), moderate (class 2, 99/444, 22.2%), high (class 3, 138/444, 31%), and very high (class 4, 157/444, 35.3%). Our adjusted multivariate probit model indicated that, compared to class 1, class 4 participants were more likely to know where to access condom use training (marginal effect [ME]=0.23; *P*<.001), external condoms (ME=0.19; *P*<.001), and HIV testing (ME=0.23; *P*<.001). We also noted gender-based differences. Men with very high DHL, compared with those with low DHL, were more likely to know where to access SRH information (ME=0.46; *P*<.001) and condom use training (ME=0.40; *P*<.050), while women with very high DHL were more likely than those with low DHL to report knowing how to access condom use training (ME=0.12, SE=0.06; *P*<.050), external condoms (ME=0.34; *P*<.001), and HIV testing (ME=0.22, SE=0.10; *P*<.050). Regarding access to sexual health products and services in the last 3 months, class 4 respondents reported higher access to condom use training (ME=0.13, SE=0.04; *P*<.001), external condoms (ME=0.14; *P*<.050), and HIV testing (ME=0.24; *P*<.050) than class 1 respondents. Gender differences showed that among men, those with very high DHL were more likely to access condom use training (ME=0.28; *P*<.010) than those with low DHL. In contrast, among women, those with very high DHL were less likely to access SRH information (ME=–0.20; *P*<.001).

**Conclusions:**

Our findings reveal a generally high level of DHL but suboptimal awareness of and access to SRH services among urban displaced youth in Kampala. Improving SRH among urban displaced populations will require gender-responsive and culturally grounded digital sexual health interventions to increase awareness of and access to sexual health products and services.

## Introduction

### Background

While digital health technologies are powerful tools for addressing health inequities in humanitarian settings, their effectiveness depends on the digital capabilities of marginalized and underserved populations. As the global forced displacement crisis intensifies, with over 120 million forcibly displaced people worldwide [1], understanding how digital health literacy (DHL) influences health outcomes has emerged as a critical priority for advancing global health equity [2-4]. For displaced persons, barriers to health care access are compounded by digital divides, with effective sexual and reproductive health (SRH) service development and implementation remaining particularly challenging because of cultural barriers, stigma, and limited service availability [5-8]. Leveraging digital health technologies to address both sexual health service inequities and digital divides represents a key opportunity to enhance the awareness of and access to sexual health products and services among marginalized and underserved populations.

Digital technologies, such as mobile phones, texting apps, and phone-based social media apps, have demonstrated increasing potential to address health disparities [5,9], particularly concerning poor sexual health outcomes among displaced youth living in informal urban settlements [6,10]. These technologies not only serve as essential tools for adolescent socialization [11-13] but also provide effective, affordable, and private delivery channels for sexual health services, particularly for youth [6,9,14], including displaced youth living in informal urban settlements who face stigma and discrimination [13,15-21].

Interventions promoting DHL are critical for increasing the reach, accessibility, engagement, and effectiveness of digital health tools, particularly among marginalized and underserved youth. DHL, a concept that evolved from eHealth (ie, a medical and public health practice supported by a web-based platform) [12], is widely defined as the ability to find, understand, and apply eHealth information to address or solve a health issue [22,23]. Studies spanning distinct contexts have demonstrated the effectiveness of DHL in promoting various physical and sexual health outcomes among marginalized and underserved populations. For example, a digital health intervention (conducted in 2012) delivered to 118 persons living with HIV/AIDS in the United States led to increased knowledge about adherence barriers, behavioral skills (eg, scheduling medications with other daily activities), and medication misconceptions [24]. Similarly, a cross-sectional study of 2300 Chinese adults aged ≥60 years found that DHL was positively associated with health-promoting behaviors, which were in turn associated with improved health-related quality of life [25].

In Uganda, DHL interventions have shown promise. For instance, an intervention study (conducted in 2017) in rural Uganda involving 385 persons living with HIV found that a patient-centered SMS text messaging app improved retention in care and appointment attendance [26]. More recently, studies among urban refugees in Uganda found that WhatsApp (Meta) delivered interventions increased COVID-19 prevention [27]. In addition, combining HIV self-testing with 2-way text messaging enhanced HIV status knowledge [10], and virtual reality tools supported mental health promotion among urban refugee youth in Kampala [28]. However, as several studies have noted, the capacity to use digital technologies may be as important to health outcomes as technology availability. For example, a study of 445 urban refugee youth (age group of 16-24 years) living in Kampala’s informal settlements found that higher DHL was associated with greater resilience and lower levels of depression [3]. Scoping reviews aimed at assessing DHL interventions for forced migrant populations are underway, emphasizing the need for culturally sensitive and enabling environments to facilitate access to eHealth resources [7].

Despite the growing evidence, few studies have examined the link between DHL and sexual health outcomes among displaced youth living in informal urban settlements in Africa. Thus, if DHL and digital interventions are to equitably support access to sexual health products and services, it is critical to explore gender-based differences in DHL and related outcomes. Existing studies examining gender-based variations in DHL have primarily focused on older adults and have yielded inconsistent findings [26,29,30]. In contrast, a study conducted in China found that, on average, men’s DHL was higher than women’s [25]. These inconsistent findings, coupled with the lack of evidence on DHL gender variations among displaced youth (whether living in East and Southern Africa or not) living in informal urban settlements, emphasize the urgent need for more research to better inform tailored interventions for this population.

### Theory and This Study

To explore the relationship between DHL and sexual health outcomes among urban displaced youth, this study leverages insights from social cognitive theory (SCT) [31]. SCT posits that human behavior results from interactions among personal, behavioral, and environmental factors. In the context of DHL, this interplay can elucidate how urban displaced youth navigate online health information environments to achieve positive sexual health outcomes. A key SCT construct, self-efficacy, refers to an individual’s belief in their ability to perform specific actions [32]. Self-efficacy helps explain why individuals adopt and sustain healthy behaviors despite challenges. For displaced youth, DHL self-efficacy may determine how effectively they seek, interpret, and act upon digital sexual health information. SCT enables researchers to examine how personal factors (digital self-efficacy), environmental factors (gender norms and information ecosystems), and behavioral factors (information-seeking actions) interact to shape sexual health awareness and access.

Guided by SCT, this study has two aims: (1) to identify distinct DHL profiles among displaced youth and (2) to assess how these profiles are associated with awareness of and access to sexual health products and services, while accounting for gender differences. These aims recognize that while technology offers rapid and private access to health information, adolescents often face functional and interpersonal challenges in effectively using online health resources. The findings will enhance the available evidence regarding DHL’s specific impact on urban displaced youth and informal settlement residents while highlighting the need for targeted interventions that leverage digital technologies to enhance health literacy and equity, ultimately addressing DHL disparities (gender-based or otherwise).

## Methods

### Participants

Between January and March 2018, we conducted a community-based cross-sectional survey of 445 displaced youth living in informal urban settlements in Kampala, Uganda. Community partners included refugee agencies (Interaid Uganda, Young Africans for Integral Development [YARID], and Tomorrow Vijana) and Ugandan government agencies (Uganda AIDS Control Program and Ministry of Health). To participate in the study, youth were required to (1) be in the age group of 16-24 years; (2) self-identify as a refugee or displaced person or have refugee or displaced parents; (3) reside in one of the five informal urban settlements in Kampala (Kabalagala, Kansanga, Katwe, Rubaga, or Nsambya); and (4) be able to provide informed consent.

### Recruitment and Data Collection Procedures

Twelve peer research assistants who self-identified as refugee or displaced adolescent girls and young women (aged 18-24 years) were trained on methods for recruiting participants, including ensuring confidentiality and administering the tablet-based survey. Participants (N=445) were recruited through peer-network sampling, a nonprobability strategy effective for engaging marginalized and underserved populations, such as displaced youth, in research. Initial participants (“seeds”), young women aged 16-24 years with strong social ties in displaced communities and diverse backgrounds (eg, socioeconomic status and education level), received study coupons and were invited to recruit 1-5 individuals from their networks. Recruited individuals could, in turn, invite 2-5 others, continuing until the sample target was reached. Peer research assistants administered tablet-based surveys in English or Swahili in locations chosen by the participants. Respondents received an honorarium of UGX 12,500 (approximately US $3.72) for completing a 35-45-minute survey.

Trained social workers were present to respond to any participant distress, and no adverse incidents were reported. All participants received a handout with psychosocial resources, and peer research assistants provided further information about Kampala’s violence prevention and response resources, including information on mental health support and postexposure prophylaxis.

### Measures

#### Outcome Variables: Awareness of and Access to Sexual Health Products and Services

We assessed participants’ (1) awareness of sexual health products and services and (2) access to sexual health products and services in the past 3 months. The products and services included SRH information, condom use training, external condoms, HIV testing, and sexually transmitted infection (STI) testing were also measured. We measured awareness of sexual health products and services with a dichotomous question:

Are you aware of where to get (1) SRH information, (2) training on condom use, (3) external condoms, (4) HIV testing and (5) STI testing near where you live?

Access to sexual health products and services was measured using a dichotomous question:

Have you accessed (1) SRH information, (2) training on condom use, (3) external condoms, (4) HIV testing and (5) STI testing in the last 3 months?

The responses for the above questions were binary, that is, yes=1 and no=0.

#### Independent Variable: DHL

DHL was measured using the eHealth Literacy Scale [23] and validated by Okumu et al [3] among a sample of displaced Ugandan youth, with a Cronbach α of 0.98. This scale is an 8-item instrument designed to evaluate participants’ combined knowledge of, comfort with, and perceived ability to find, evaluate, and apply digital health information to address health problems. Scale items include statements such as, “I know what health resources are available on the Internet” and “I feel confident in using information from the Internet to make health decisions.” These items collectively evaluate the respondents’ awareness of available health resources, their ability to locate and use these resources, and their confidence in discerning the quality of the information found. The 8 items were rated on a 5-point Likert scale, ranging from 1 (strongly disagree) to 5 (strongly agree), and were used to identify the digital capabilities of young people using latent class analysis.

Sociodemographic variables included age (continuous), gender (binary; men or women), education level (categorical: no education, below secondary, secondary, and tertiary), employment status (categorical: employed, unemployed, and student), time in Uganda (categorical: <1 year to >5 years), and relationship status (categorical: no relationship, dating one partner or married, and casual dating or multiple partners).

#### Data Analysis

We first conducted descriptive analyses of all variables to determine the frequencies and proportions for categorical variables and the means and SDs for continuous variables. To address the first aim, identifying distinct DHL profiles, we performed latent profile analysis using maximum likelihood. Following best practices, we started with a 1-class solution and iteratively tested models with additional classes. Model fit was evaluated using the Akaike information criterion (AIC; [33]), Bayesian information criterion (BIC; [34]), and BIC value adjusted for sample size (ABIC; [35]). As BIC is the most reliable of these information criterion indices, with lower BIC values indicating a good model fit, we assessed the sensitivity of BIC scores across models. We also calculated an entropy score for each model to determine how well the indicators represented class membership, with higher entropy scores (ie, closer to 1) indicating better class representation [36]. In determining the optimal number of classes, we also sought to ensure that no profile contained a disproportionately small number of participants (ie, less than 5% of all respondents). Finally, we ensured that the pattern of results for each profile made theoretical sense [37] by inspecting the mean score for each variable.

To address the second aim, we used the multivariate probit technique to simultaneously estimate participants’ probability of gaining awareness of all 5 sexual health products and services (ie, SRH information, condom use training, external condoms, HIV testing, and STI testing) conditioned on the same set of explanatory variables. We used the same method to estimate each participant’s probability of accessing all 5 sexual health products and services. We used the multivariate probit model in our analysis because we assumed that an individual’s awareness of and access to a set of sexual health products and services were not mutually exclusive but usually occur simultaneously in practice. We then calculated the marginal effects in percentage points for each association.

### Ethical Considerations

Ethical approval was granted by the University of Toronto (#35,405), Toronto, Ontario, and the Ministry of Health of Uganda (ADM: 105/261/01). The study was authorized by the Office of the Prime Minister of Uganda. All participants provided electronic informed consent before enrollment. Data security and confidentiality for the tablet-based surveys were ensured through the automatic encryption of all collected data and daily uploads to a password-protected project server using Secure Sockets Layer. Participants were assigned unique case IDs, and no personally identifiable information was stored with the study data. These procedures were consistent with institutional data protection guidelines and ethics approvals

## Results

### Sample Characteristics

As shown in Multimedia Appendix 1, over half of the participants were adolescents (243/445, 54.61%) aged 16-19 years. More than two thirds of the participants identified as women (333/445, 74.83%), and approximately 47.42% (211/445) had completed secondary education. For digital technologies, most participants owned and used mobile phones (331/445, 74.4%), sent an average of 3.46 (SD 1.93) texts per day, and used an average of 3.07 (SD 1.90) mobile apps (eg, Facebook [Meta], WhatsApp [Meta], and email) concurrently.

#### Sexual Health Equity Among Displaced Youth Living in the Informal Settlements of Kampala, Uganda

As illustrated in Table 1 shows the discrepancies between participants’ awareness and 3-month access to sexual health products and services (ie, SRH, condom use training, external condoms, STI, and HIV testing). For instance, 29.1% (129/444) of participants reported awareness of how to access SRH information, but only 14% (62/444) reported actually accessing SRH information. Regarding condom use training, 15.3% (68/444) reported awareness of where to access the training, whereas only 5.6% (25/444) reported accessing the training. For testing, 47.5% (211/444) reported that they were aware of where to access HIV testing, but only 25.9% (115/444) reported accessing HIV testing services. Similarly, 23.6% (105/444) reported awareness of how to access STI testing, and only 11.9% (53/444) reported accessing STI testing in the previous 3 months.

**Table 1:** Sexual health equity outcomes among displaced youth in the slums of Kampala, Uganda (N=444).

Outcome	Having awareness, n (%)	Not having awareness, n (%)	Having access, n (%)	Not having access, n (%)
SRH information	129 (29.1)	315 (70.9)	62 (14)	382 (86)
Condom use training	68 (15.3)	376 (84.7)	25 (5.6)	419 (94.4)
External condoms	245 (55.1)	200 (44.9)	109 (24.5)	336 (75.5)
HIV testing	211 (47.5)	233 (74.1)	115 (25.9)	329 (74.1)
STI testing	105 (23.6)	339 (76.4)	53 (11.9)	391 (88.1)

#### Four-Class Solution for DHL

**Figure 1:**
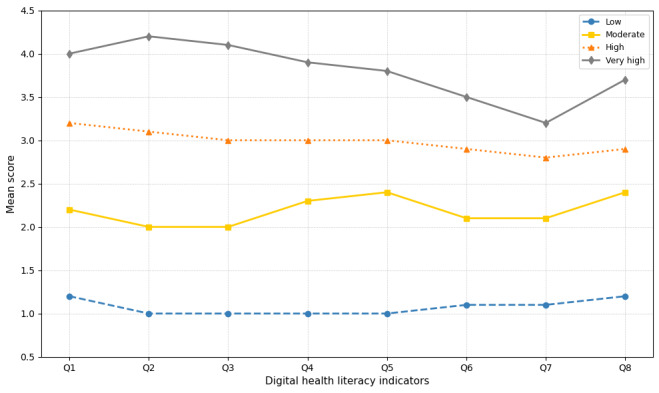
Digital health literacy profiles among displaced youth in informal urban settlements of Kampala, Uganda (N=444).

To answer our first question, we conducted a latent profile analysis and compared the model fit indices, number of parameters, and classification errors for models with 1-6 clusters (Multimedia Appendix 2). BIC and AIC values continued to decrease as the number of classes (K) increased; however, this improvement was progressively smaller after 3 classes (Figure 1). Based on the interpretability of the latent profiles, the reduction in class size beyond K=4, and parsimony, the 4-class model was selected as the optimal class structure. The 4-cluster model’s entropy value was 0.98.

Class 1, named the low DHL group (51/444, 11.46%), exhibited low levels for all 8 indicators (Figure 1). Class 2 was named the moderate DHL group (99/444, 22.25%). Class 3 was named the high DHL group (138/444, 31.01%). The final, class 4, was named the very high DHL group (157/444, 35.28%), because it exhibited high values for all 8 indicators.

#### Digital Health Literacy’s Association With Awareness of Sexual Health Products and Services

Table 2 shows the association between DHL and awareness of where to access sexual health products and services. In respective terms, respondents with moderate and very high DHL have 21% and 23% higher probabilities of being aware of where to access HIV testing services than those with low DHL. Moreover, a person with very high DHL has a 23% higher probability of being aware of where to access condom use training and a 19% higher probability of being aware of where to access external condoms compared to someone with low DHL.

**Table 2:** Digital health literacy and awareness of sexual health products and services (main model: N=444).

Variable			SRH^a^ information		Condom use training		External condoms		STI^b^ testing		HIV testing
**Digital health literacy (base: low), marginal effect (SE)**											
	Moderate	–0.01 (0.08)		0.10 (0.06)		0.00 (0.07)		0.07 (0.08)		0.2^c^ (0.09)	
	High	–0.04 (0.07)		0.05 (0.06)		0.11 (0.06)		0.03 (0.07)		0.15 (0.08)	
	Very high	0.10 (0.08)		0.23^d^ (0.06)		0.19^d^ (0.07)		0.10 (0.08)		0.23^d^ (0.09)	
Women, marginal effect (SE)			–0.15^d^ (0.05)		–0.25^d^ (0.04)		–0.37^d^ (0.04)		0.00 (0.05)		–0.10^e^ (0.05)
Age, marginal effect (SE)			0.04^d^ (0.01)		0.02^d^ (0.01)		0.06^d^ (0.01)		0.08^d^ (0.01)		0.02^e^ (0.01)
Dating, marginal effect (SE)			–0.01 (0.05)		0.04 (0.04)		0.19^d^ (0.05)		–0.01 (0.05)		0.07 (0.06)
Mobile phone ownership, marginal effect (SE)			–0.05 (0.06)		–0.09^e^ (0.04)		0.06 (0.05)		0.02 (0.06)		–0.00 (0.06)
Economic insecurity, marginal effect (SE)			–0.05 (0.05)		–0.04 (0.04)		–0.01 (0.04)		0.06 (0.05)		0.07 (0.05)
**Time in Uganda (base: <1 year), marginal effect (SE)**											
	Between 1-5 years	–0.03 (0.08)		0.06 (0.06)		0.20^d^ (0.07)		0.14^c^ (0.08)		0.18^e^ (0.08)	
	More than 5 years	–0.06 (0.08)		0.11^c^ (0.06)		0.10 (0.07)		0.12 (0.08)		0.16^c^ (0.09)	
**Education (base: tertiary), marginal effect (SE)**											
	No education	0.07 (0.10)		0.09 (0.08)		0.25^d^ (0.09)		0.36^d^ (0.11)		0.17 (0.11)	
	Below secondary	–0.02 (0.08)		0.04 (0.06)		–0.02 (0.07)		0.16^e^ (0.08)		–0.24^d^ (0.08)	
	Secondary level	–0.02 (0.07)		0.03 (0.05)		0.10^c^ (0.06)		0.00 (0.07)		–0.08 (0.07)	
Observations, n			444		444		444		444		444

^a^SRH: sexual and reproductive health.

^b^STI: sexually transmitted infection.

^c^*P*<.050.

^d^*P*<.001

^e^*P*<.010.

#### Gender-Based Analysis

Compared to a man with low DHL, a man with very high DHL has a 46% higher probability of being aware of SRH information and a 40% higher probability of being aware of available condom use training (see Panel A, Table 3). Compared to a woman with low DHL, a woman with very high DHL has a 12% higher probability of being aware of available condom use training and a 22% higher probability of being aware of HIV testing service locations. Furthermore, compared to women with low DHL, women with high and very high DHL have a 20% and 34% higher probability, respectively, of being aware of the availability of external condoms (see Panel B, Table 3).

**Table 3:** Digital health literacy and awareness on how to access sexual health products and services by gender (N=444).

Variable			SRH^a^ information	Condom use training	External condoms	STI^b^ testing	HIV testing
**Panel A: men sample**							
	**Digital health literacy (base=low), marginal effect (SE)**						
		Moderate	0.26 (0.17)	0.23 (0.17)	–0.06 (0.10)	–0.06 (0.16)	0.23 (0.17)
		High	0.30 (0.17)	0.05 (0.16)	–0.03 (0.10)	–0.19 (0.16)	0.30 (0.17)
		Very high	0.46^d^ (0.16)	0.40^c^ (0.16)	–0.16 (0.09)	0.03 (0.15)	0.29 (0.16)
	All other controls		Yes	Yes	Yes	Yes	Yes
	Observations, n		112	112	112	112	112
**Panel B: women sample**							
	**Digital health literacy (base=low), marginal effect (SE)**						
		Moderate	–0.10 (0.09)	0.02 (0.06)	0.04 (0.09)	0.07 (0.09)	0.16 (0.11)
		High	–0.14 (0.08)	0.02 (0.05)	0.20^d^ (0.08)	0.06 (0.08)	0.10 (0.10)
		Very high	–0.05 (0.09)	0.12^c^ (0.06)	0.34^d^ (0.09)	0.09 (0.09)	0.22^c^ (0.10)
	All other controls		Yes	Yes	Yes	Yes	Yes
	Observations, n		332	332	332	332	332

^a^SRH: sexual and reproductive health.

^b^STI: sexually transmitted infection.

^c^*P*<.050.

^d^*P*<.001.

^e^*P*<.010.

#### Digital Health Literacy’s Association With Recent Access of Sexual Health Products and Services

Table 4 presents data on the relationship between DHL and recent access to sexual health products and services. Across the board, respondents with moderate and very high DHL had a 24% higher probability of accessing HIV testing than those with low DHL. Furthermore, participants with very high DHL had a 13% higher probability of having access to condom use training and a 14% higher probability of having access to external condoms compared to those with low DHL. Respondents with high DHL had, on average, a 14% lower probability of accessing SRH information than those with low DHL.

**Table 4:** Digital health literacy and recent access of sexual health products and services (main model: N=444).

Variable		SRH^a^ information	Condom use training	External condoms	STI^b^ testing	HIV testing
**Digital health literacy (base: low), marginal effect (SE)**						
	Moderate	–0.10 (0.06)	0.05 (0.04)	0.03 (0.07)	0.07 (0.08)	0.24^c^ (0.11)
	High	–0.14^c^ (0.06)	0.07 (0.04)	0.10 (0.07)	0.03 (0.07)	0.09 (0.10)
	Very high	–0.10 (0.06)	0.13^d^ (0.04)	0.14^c^ (0.07)	0.10 (0.08)	0.24^c^ (0.10)
Women, marginal effect (SE)		0.01 (0.04)	–0.11^d^ (0.03)	–0.17^d^ (0.04)	0.00 (0.05)	0.12^c^ (0.05)
Age, marginal effect (SE)		0.02^c^ (0.01)	0.01^c^ (0.01)	0.04^d^ (0.01)	0.08^d^ (0.01)	0.02 (0.01)
Dating, marginal effect (SE)		–0.03 (0.04)	0.01 (0.03)	0.16^d^ (0.05)	–0.01 (0.05)	0.03 (0.05)
Mobile phone ownership, marginal effect (SE)		0.03 (0.04)	–0.11^d^ (0.03)	–0.07 (0.05)	0.02 (0.06)	0.04 (0.06)
Economic insecurity, marginal effect (SE)		–0.06 (0.04)	–0.01 (0.02)	0.08^c^ (0.04)	0.06 (0.05)	0.05 (0.05)
**Time in Uganda (base: <1 year), marginal effect (SE)**						
	Between 1-5 years	0.08 (0.06)	–0.01 (0.04)	0.15 (0.07)	0.14 (0.08)	0.14 (0.08)
	More than 5 years	0.07 (0.07)	0.01 (0.04)	0.17 (0.07)	0.12 (0.08)	0.05 (0.08)
**Education (base: tertiary), marginal effect (SE)**						
	No education	0.10 (0.08)	0.13 (0.05)	0.06 (0.09)	0.36^d^ (0.11)	0.30^d^ (0.11)
	Below secondary	0.02 (0.06)	0.06 (0.04)	0.08 (0.07)	0.16 (0.08)	–0.06 (0.08)
	Secondary level	–0.01 (0.05)	0.04 (0.04)	0.15 (0.06)	0.00 (0.07)	0.00 (0.06)
Observations, n		444	444	444	444	444

^a^SRH: sexual and reproductive health.

^b^STI: sexually transmitted infection.

^c^*P*<.050.

^d^*P*<.001.

^e^*P*<.010.

#### Gender-Based Analysis

Among men, those with very high DHL had a 28% higher probability of accessing condom use training compared to those with low DHL (Panel A, Table 5). Compared with women with low DHL, those with moderate, high, and very high DHL had 17%, 19%, and 20% lower probabilities, respectively, of accessing SRH information (Panel B, Table 5).

**Table 5:** Digital health literacy and recent access to sexual health products and services by gender (N=444).

Variable			SRH^a^ information	Condom use training	External condoms	STI^b^ testing	HIV testing
**Panel A: men sample**							
	**Digital health literacy (base=low), marginal effect (SE)**						
		Moderate	0.06 (0.13)	0.11 (0.12)	0.19 (0.17)	–0.06 (0.16)	0.06 (2.15)
		High	–0.05 (0.13)	0.12 (0.12)	0.23 (0.17)	–0.19 (0.16)	1.00 (1.99)
		Very high	0.09 (0.12)	0.28^e^ (0.11)	0.28 (0.16)	0.03 (0.15)	1.15 (2.24)
	All other controls		Yes	Yes	Yes	Yes	Yes
	Observations, n		112	112	112	112	112
**Panel B: women sample**							
	**Digital health literacy (base=low), marginal effect (SE)**						
		Moderate	–0.17^c^ (0.07)	0.01 (0.04)	–0.01 (0.08)	0.07 (0.09)	0.18 (0.13)
		High	–0.19^e^ (0.07)	0.02 (0.03)	0.05 (0.07)	0.06 (0.08)	–0.01 (0.13)
		Very high	–0.20^d^ (0.07)	0.05 (0.04)	0.08 (0.08)	0.09 (0.09)	0.15 (0.13)
	All other controls		Yes	Yes	Yes	Yes	Yes
	Observations, n		332	332	332	332	332

^a^SRH: sexual and reproductive health.

^b^STI: sexually transmitted infection.

^c^*P*<.050.

^d^*P*<.001.

^e^*P*<.010.

## Discussion

### Principal Findings

This study produced multiple novel insights, revealing distinct DHL profiles and associations between DHL and awareness of and access to sexual health products and services. We also found gender-based differences in how DHL influences sexual health outcomes. These findings highlight the critical role of digital literacy as a social determinant of health in humanitarian contexts. Collectively, these findings highlight the need for contextualized, gender-specific interventions to advance digital determinants of sexual health equity among forcibly displaced populations.

We identified 4 distinct DHL profiles—low (11.5%), moderate (22.2%), high (31%), and very high (35.3%)—a significant finding that calls into question the applicability of a binary “digital divide” among displaced populations [3,38]. This finding is corroborated by Veinot et al [39], who argued that digital inequality manifests across multiple domains of digital access, skills, and engagement. The relatively large proportion of respondents with high or very high DHL (295/444, 66.3%) challenges the assumption of deficit-oriented narratives about universally low digital literacy among displaced populations. More recent studies have documented considerable digital capabilities among urban displaced youth, who leverage digital tools to enhance their health agency despite structural barriers to connectivity [3,10,13,27,28]. Our findings align with recent calls for more tailored DHL-related sexual health interventions [8] by providing a precision-oriented approach to intervention programming matched to an individual’s or community’s DHL levels. For example, the 11.5% (51/444) of displaced youth in our sample who were classified as having low DHL will require intensive support focused on basic digital navigation skills, while those with moderate to high DHL may benefit from more specialized training focused on evaluating digital health information. This tailored approach adheres to and extends the World Health Organization’s (WHO) [4] emphasis on the need for context-specific digital health interventions that account for, rather than presume, clients’ existing capabilities.

Our observed association between very high DHL and increased awareness of sexual health products and services, particularly condom use training, external condoms, and HIV testing, underscores the importance of digital literacy in increasing awareness to sexual health information for HIV prevention. Compared to respondents with low DHL, those with very high DHL demonstrated a 21%-23% higher probability of being aware of HIV testing services, highlighting the potential of digital sexual health interventions to address critical gaps in sexual health knowledge among displaced populations. This finding exemplifies what Bandura [32] termed self-efficacy’s “knowledge acquisition function,” whereby stronger self-efficacy beliefs motivate an individual’s information-seeking behaviors and enhance their cognitive processing of health information. Indeed, a recent scoping review of 57 studies [40] found that digital health interventions increased awareness of available health services among refugee populations. DHL likely enhances awareness of sexual health products and services by improving individuals’ ability to navigate online sexual health resources, assess the quality of sexual health information, and engage with digital sexual health platforms—skills that are critical for facilitating access to health information among displaced populations [3].

We also observed gender-based differences in sexual health service awareness patterns, highlighting the inextricability of digital technologies from existing gender norms and information-seeking behaviors. Men with very high DHL reported greater awareness of SRH information and condom use training, whereas women with very high DHL reported greater awareness of condom use training, external condoms, and HIV testing. These patterns align with qualitative findings on sexual health literacy that refugee men and women (18-24 years) access sexual health information through different information ecologies, with men preferring peers, teachers, and online sources. In contrast, women increasingly rely on their parents for sexual health information [41]. Furthermore, distinct sexual health service awareness patterns may reflect gendered responsibilities for sexual health, with women often bearing greater responsibility for contraception and HIV prevention in heterosexual relationships [6,13,15-18,42]. While high digital health literacy may enhance women’s self-efficacy, entrenched environmental constraints, such as restrictive gender norms, social surveillance, and limited autonomy in decision-making, can significantly inhibit the translation of knowledge into preventive behaviors. For example, even when young women are well-informed about condom use or HIV testing, cultural expectations surrounding female sexuality, fear of judgment, and partner disapproval may discourage them from acting on this knowledge [5,13,43]. Indeed, Bandura’s [31] SCT emphasizes that behavior is not solely a function of knowledge or personal agency but is also shaped by environmental enablers and barriers. These findings underscore the need for gender-sensitive digital sexual health interventions that address the reciprocal relationship between personal factors (digital self-efficacy), behavioral patterns (information seeking), and environmental influences (gender norms and information ecosystems) [31,32].

We also observed a disconnect between awareness of and the likelihood of accessing sexual health products and services, particularly among women with high digital health literacy. Specifically, respondents with very high DHL generally reported higher access to condom use training and external condoms than those with low DHL. However, gender-stratified analysis revealed that women with very high DHL were less likely to access SRH information than those with low DHL. The fact that women with very high DHL did not appear as likely as men with very high DHL to access sexual health products and services exemplifies how environmental constraints faced by refugee women—such as inequitable gender norms [13,16], mobility constraints [15,44], and adolescent SRH stigma [16,17,21]—can prevent the translation of digital knowledge into service usage despite sufficient knowledge and skills [31,32]. Women refugees often face heightened vulnerability to cyber harassment and privacy violations [6,21]. A prior Ugandan study showed that men participated twice as much as women in an SMS text messaging–based HIV campaign [45]. Women are also disproportionately excluded from shaping digital system infrastructure, which could promote DHL and engagement with services [46]. From an SCT perspective, lower engagement stemming from privacy concerns illustrates how environmental threats can undermine the motivational effects of self-efficacy when individuals perceive the risks associated with service engagement as substantial [31]. This complex interplay between DHL and gender-specific constraints highlights the need for interventions that support technical skill development while considering the broader socioecological context in which these skills are applied. Thus, digital sexual health interventions focused solely on enhancing technical skills may inadvertently widen gender gaps unless they address the sociostructural constraints that disproportionately affect women.

### Implications for Intervention and Policy

The findings of this study have multiple implications for designing theoretically grounded interventions aimed at enhancing DHL and promoting sexual health equity among displaced youth in Uganda.

First, we recommend co-designing digital health platforms with displaced communities to ensure that these tools are user-friendly, accessible, and relevant to their experiences [47]. This collaborative approach enhances digital health literacy, fostering user trust and engagement. Recent studies on DHL emphasize that integrating gamification and social support into digital interventions can sustain engagement and improve health outcomes [47,48]. By combining these insights, we can develop comprehensive programs that leverage digital technologies to improve SRH outcomes for displaced youth while respecting their cultural and contextual requirements. Given our observation that, particularly for women, awareness of available digital services does not automatically translate into access to those services, the goal of promoting DHL should be integrated into broader health equity frameworks and addressed alongside other social determinants of health, such as gender, education, and economic status.

Second, given our findings, it is critical that interventions match participants’ DHL levels while also addressing contextual barriers. For instance, the Tushirikiane projects with displaced youth in Kampala, Uganda, co-developed and implemented low (comic books) [49,50]and high (2-way text messages and WhatsApp groups) tech digital interventions [10,27,28], highlighting the importance of addressing social norms and stigma that hinder access to SRH information and services. Furthermore, online pharmacies, such as Rocket Health, which have increased access to SRH self-care products among youth in Uganda, can be leveraged to provide confidentiality to mitigate stigma and increase access to SRH information [51]. Despite limited research on the online pharmacy engagement of displaced youth, studies on community pharmacies highlight their potential as an existing infrastructure for delivering SRH services, including STI screening and contraception, both of which are highly valued by users [52]. For displaced youth, online platforms can complement traditional health care services by offering confidential channels for accessing SRH information and products. This confidential access can be particularly beneficial in settings where access to physical health care infrastructure is limited or is stigmatized. In addition, integrating digital literacy training with peer navigation support has been shown to empower displaced youth to critically evaluate health information and advocate for their needs [15]. This integrated approach can help bridge the gap between displaced youth’s awareness of and access to SRH products and services by providing tailored support and resources that address the unique challenges faced by displaced youth in Uganda.

Finally, we suggest integrating digital interventions with traditional service delivery models. Uganda’s Health Sector Integrated Refugee Response Plan provides a framework for integrating comprehensive primary health care services for refugees into the national health system [53]. Building on this framework, digital health interventions can complement traditional health care services by offering confidential and accessible channels for SRH information and services to young people. For instance, developing mobile apps or GPS-enabled platforms that provide SRH literacy and resources can help reduce stigma and increase access to care, particularly in settings where physical access to care is limited.

### Limitations

The novel findings of this study should be interpreted in light of several limitations. The cross-sectional design of our study limits our ability to establish causal relationships between DHL and sexual health service awareness or access. Future longitudinal research would be better equipped to analyze how changes in DHL affect sexual health service usage. Another limitation is the peer network recruitment method, which may have led to increased participation by young people who were digitally active. This could have biased the DHL results. Future studies should use strategies to diversify digital engagement among youth. Although stigma and gender norms are frequently identified as obstacles to service usage, our study did not gather direct data on these factors. Further research is necessary to investigate the underlying causes of the “awareness versus access” gap among youth. In addition, as our study focused on displaced youth in urban informal settings in Kampala, Uganda, our findings may not be generalizable to other refugee or displacement contexts with different technological infrastructures and cultural norms. Finally, our study relied on self-reported data, which can be particularly vulnerable to social desirability. Despite these limitations, our use of latent class analysis to identify distinct DHL profiles represents a methodological strength that enables a more precise measurement and conceptualization of DHL. Therefore, future research should explore the longitudinal impact of digital health literacy interventions and examine how intersecting factors (eg, age, education, and displacement duration) may further moderate the relationship between digital literacy and sexual health outcomes. In addition, mixed methods studies that combine quantitative DHL profile assessments with a qualitative exploration of barriers and facilitators to service usage would provide deeper insight into the mechanisms underlying the gender-based differences observed in our study.

### Conclusion

Our study demonstrates that DHL functions as a significant determinant of sexual health equity among displaced youth in Kampala, Uganda, and that this relationship is nuanced and moderated by gender. The 4 identified DHL profiles provide a framework for tiered interventions targeting clients with varying digital capabilities. DHL’s differential impact on service awareness versus service access, particularly among women, highlights the need for intervention designs that address both technical skills and structural barriers to service usage. As digital sexual health interventions continue to expand in humanitarian settings, ensuring equity in the digital capabilities of service populations and translating these capabilities into improved sexual health outcomes are urgent priorities. By approaching DHL as a social determinant of sexual health and addressing the complex interplay between digital skills, gender norms, and structural barriers in a service context, digital sexual health initiatives can meaningfully advance sexual health equity among the 2 million refugees in Uganda and over 123 million displaced persons globally.

## Appendix

Multimedia Appendix 1 Characteristics of forcibly displaced youth living in informal settlements in Kampala, Uganda (N=445).

Multimedia Appendix 2 Latent class enumeration statistics of digital health literacy of displaced youth living in the informal settlements of Kampala, Uganda.

## Abbreviations

AIC: Akaike information criterion
BIC: Bayesian information criterion
DHL: digital health literacy
SCT: social cognitive theory
SRH: sexual and reproductive health
STI: sexually transmitted infection
WHO: World Health Organization
YARID: Young Africans for Integral Development

## Data Availability

The datasets analyzed during the current study are not publicly available due to ethical and privacy considerations related to the protection of refugee participants. However, deidentified data may be available from the corresponding author upon reasonable request and with appropriate institutional approvals.
