# Supplementary material for: Digital Health Literacy and Its Role in Awareness of and Access to Sexual Health Products and Services Among Displaced Youth in Uganda’s Informal Urban Settlements: Community-Based Cross-Sectional Study
Source: J Med Internet Res. 2025 Dec 31;27:e78343. doi: 10.2196/78343 (PMC12805323; doi:10.2196/78343)
Supplement: Multimedia Appendix 1 [file jmir_v27i1e78343_app1.docx]

**Multimedia Appendix 1.** Characteristics of forcibly displaced youth living in informal settlements in Kampala, Uganda (N=445)

| Variables | | Values |
| --- | --- | --- |
| **Sociodemographic factors** | | |
| **Age**, **n (%)** | | |
|  | Adolescents | 243 (54.61) |
|  | Young adults | 202 (45.39) |
| **Gender, n (%)** | | |
|  | Women | 333 (74.83) |
|  | Men | 112 (25.17) |
| **Education, n (%)** | | |
|  | No education | 42 (9.44) |
|  | Less than secondary school | 192 (43.15) |
|  | Secondary education | 148 (33.26) |
|  | Tertiary education | 63 (14.16) |
| **Time in Uganda (year), n (%)** | | |
|  | <1 | 300 (67.42) |
|  | 6-10 | 102 (22.92) |
|  | >10 | 43 (9.66) |
| **Employment status, n (%)** | | |
|  | Employed | 252 (58.88) |
|  | Unemployed | 176 (41.12) |
| **Digital technology use** | | |
| **Mobile phone ownership and use** | | |
|  | No | 114 (25.6) |
|  | Yes | 331 (74.4) |
|  | Average text messages sent per day | 3.46 (1.93, 1-7) |
|  | Average mobile app usage | 3.07 (1.90, 0-9) |
