# Supplementary material for: Digital Health Literacy and Its Role in Awareness of and Access to Sexual Health Products and Services Among Displaced Youth in Uganda’s Informal Urban Settlements: Community-Based Cross-Sectional Study
Source: J Med Internet Res. 2025 Dec 31;27:e78343. doi: 10.2196/78343 (PMC12805323; doi:10.2196/78343)
Supplement: Multimedia Appendix 2 [file jmir_v27i1e78343_app2.docx]

**Multimedia Appendix 2.** Latent class enumeration statistics of DHL of displaced youth living in the informal settlements of Kampala, Uganda

| Class | Loglikelihood | AIC^a^ | BIC^b^ | SABIC^c^ | Entropy | LMR^d^ P value |
| --- | --- | --- | --- | --- | --- | --- |
| 1 | –8125.863 | 16287.726 | 16361.491 | 16304.367 | —^e^ | — |
| 2 | –7170.804 | 14397.608 | 14512.354 | 14423.494 | 0.956 | 0.001 |
| 3 | –6797.802 | 13671.605 | 13827.332 | 13706.736 | 0.946 | 0.001 |
| 4 | –6557.572 | 13211.145 | 13407.853 | 13255.521 | 0.981 | 0.078 |
| 5 | –6339.771 | 12795.541 | 13033.230 | 12849.162 | 0.972 | 0.018 |

Notes.

^a^AIC: Akaike information criterion.

^b^BIC: Bayesian information criterion.

^c^SABIC: Sample size-adjusted Bayesian information criterion.

^d^LMR: Lo-Mendell-Rubin.

^e^Not applicable.
